# External validation of the R.I.R.S. scoring system to predict stone-free rate after retrograde intrarenal surgery

**DOI:** 10.1186/s12894-021-00801-y

**Published:** 2021-03-04

**Authors:** Cong Wang, ShouTong Wang, Xuemei Wang, Jun Lu

**Affiliations:** 1 Department of Urology, Shanghai General Hospital, Nanjing Medical University, No.100, Haining Road, Hongkou District, Shanghai, 200080 China; 2 Department of Urology, Shanghai General Hospital, Shanghai Jiao Tong University School of Medicine, No.100, Haining Road, Hongkou District, Shanghai, 200080 China

**Keywords:** Urolithiasis, Retrograde intrarenal surgery, Prognosis, Scoring system

## Abstract

**Background:**

The R.I.R.S. scoring system is defined as a novel and straightforward scoring system that uses the main parameters (kidney stone density, inferior pole stones, stone burden, and renal infundibular length) to identify most appropriate patients for retrograde intrarenal surgery (RIRS). We strived to evaluate the accuracy of the R.I.R.S. scoring system in predicting the stone-free rate (SFR) after RIRS.

**Methods:**

In our medical center, we retrospectively analyzed charts of patients who had, between September 2018 and December 2019, been treated by RIRS for kidney stones. A total of 147 patients were enrolled in the study. Parameters were measured for each of the four specified variables.

**Results:**

Stone-free status was achieved in 105 patients (71.43%), and 42 patients had one or more residual fragments (28.57%). Differences in stone characteristics, including renal infundibulopelvic angle, renal infundibular length, lower pole stone, kidney stone density, and stone burden were statistically significant in patients whether RIRS achieved stone-free status or not (*P* < 0.001, *P*: 0.005, *P* < 0.001, *P* < 0.001, *P*: 0.003, respectively). R.I.R.S. scores were significantly lower in patients treated successfully with RIRS than patients in which RIRS failed (*P* < 0.001). Binary logistic regression analyses revealed that R.I.R.S. scores were independent factors affecting RIRS success (*P* = 0.033). The area under the curve of the R.I.R.S. scoring system was 0.737.

**Conclusions:**

Our study retrospectively validates that the R.I.R.S. scoring system is associated with SFR after RIRS in the treatment of renal stones, and can predict accurately.

## Background

With the continuous improvement of flexible ureteroscope equipment, such as endoscope miniaturization, improved deflection angles, enhanced optical quality, and ancillary tools, more and more urologists have become inclined to use retrograde intrarenal surgery (RIRS) to treat kidney stones [1, 2]. Although the current urolithiasis guidelines recommend RIRS for renal stones smaller than 20 mm, with the accumulation of experience of the surgeon and the preoperative judgment of the difficulty of the operation, more surgeons have engaged RIRS to resolve renal stones larger than 20 mm and achieved excellent results [3, 4].

The ensuing question is how to choose the surgical method before surgery, RIRS or percutaneous nephrolithotomy (PCNL), and the postoperative stone-free rate (SFR) is a significant factor. To solve this problem, in 2017, Xiao et al. published the R.I.R.S. scoring system with four different parameters: kidney stone density, inferior pole stones, stone burden, and renal infundibular length (RIL). In this system, each parameter is assigned a different point according to different situations. The total score can range from 4 to 10 points. The higher the score, the more complicated the stone. Although the R.I.R.S. scoring system showed reliable predictive power in the preliminary research results, and there is still a lack of retrospective validation. The purpose of this article is to validate this scoring system and evaluate its validity [5].

## Methods

**Fig. 1:**
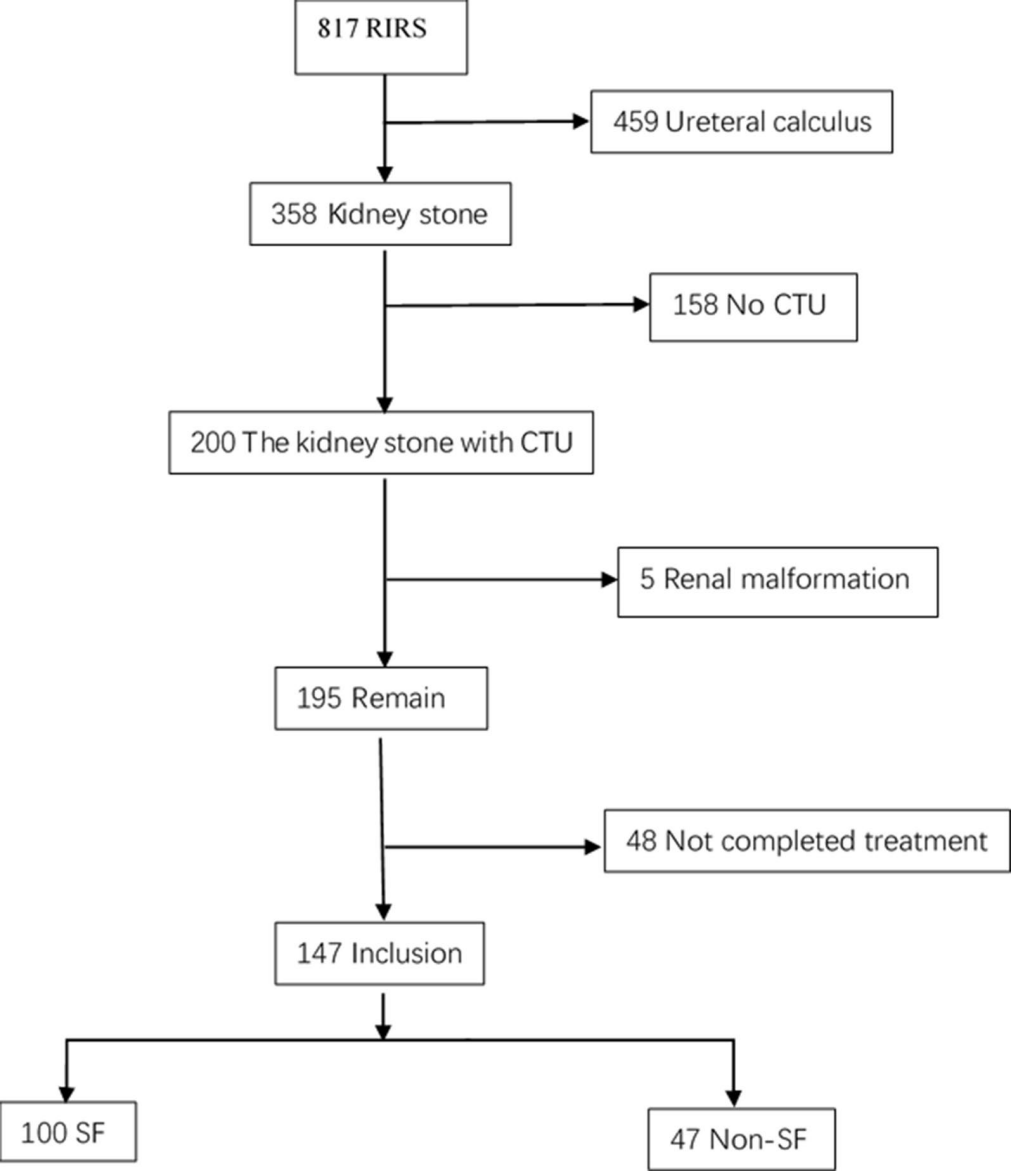
Flowchart of the enrolled patients

This retrospective study was supported by our Institutional Review Board (IRB No. 2018KY192-3). At the urology department of Shanghai General Hospital, case data from patients undergoing RIRS treatment for kidney stones between September 2018 and December 2019 were retrospectively analyzed. A total of 158 patients who had not been evaluated by computed tomography urography (CTU) before the RIRS and 48 patients who had not completed treatment were excluded. Other exclusion criteria were pelviureteric mass and musculoskeletal or renal malformation. At the end of the evaluation, a total of 147 patients were enrolled in the study (Fig. 1).

In our study, all patients were evaluated by CTU before the RIRS. Patients were evaluated with a kidney ureter bladder (KUB) X-ray or non-contrast computed tomography (NCCT) 1 month after RIRS. Stone-free status was defined as no detectable stone on KUB or NCCT, or fragments of less than 2 mm were also deemed negligible stones [6]. The patients’ preoperative characteristics were recorded. These were including age, gender, hypertension, diabetes mellitus status, body mass index (BMI), urine culture, history of surgery, stone location, and laterality of each stone. CTU also assessed number of stones, renal stone density, inferior pole stone, RIL, RIW, RIPA and stone burden. We divided the patients into three groups by their R.I.R.S. scores: 4–5 was mild, 6–8 was moderate, and 9–10 was severe. Using the data we collected, we also calculated the Resorlu-Unsal Stone Score (RUSS) score.

### Patients and procedures

All patients completed relevant examinations before surgery to exclude contraindications to operation. All patients were treated with single-dose antibiotics before RIRS. Sensitive antibiotics were used in patients with positive urine culture until negative. An experienced urologist performed all surgeries in the lithotomy position under general anesthesia. All patients were treated with a ureteral access sheath during the operation, promoting the removal of stones and reducing the renal pressure [7]. A flexible ureteroscope was then used for holmium laser lithotripsy via ureteral access sheath (UAS), and the fragments were removed using a stone extractor [8]. When the operation took longer than 90 min, it was stopped to prevent complications. A double j-stent was placed after the operation and was removed about postoperative 1 month.

### Data and statistical analysis

Data analysis was accomplished using the SPSS statistical software package (v25.0; IBM, US). Continuous variables were expressed as mean and standard deviation (SD) or median (Q3–Q1; interquartile range); categorical variables were expressed as percentages. Continuous variables were examined using a Student’s t test or Mann–Whitney U test. Chi-square test and Fisher's exact test were used to compare the pairs of categorical variables. The two-tailed *P* value < 0.05 was considered statistically significant. Binary logistic regression analysis was used to identify independent predictors of stone-free status after RIRS treatment. We evaluated the predictive ability of the R.I.R.S. scoring system based on the area under the curve (AUC) from the receiver operating characteristic (ROC).

## Results

147 patients participated in this study. 105 (71.43%) achieved stone-free status, and 42 patients (28.57%) had one or more residual fragments. The age, gender, mean BMI, and laterality of stones were similar between the groups (*P*: 0.494, *P*: 0.949, *P*: 0.139, *P*: 0.085, respectively). The stone characteristics such as renal infundibulopelvic angle (RIPA), inferior pole stone, RIL, kidney stone density, and stone burden have statistically significant differences whether RIRS achieved stone-free status or not (*P* < 0.001, *P* < 0.001, *P*: 0.005, *P* < 0.001, *P*: 0.003). Compared with patients who failed RIRS, patients who successfully applied RIRS had a significantly lower scoring system (*P* < 0.001). Preoperative patient characteristics and stone parameters are shown in Table 1. The SFR was significantly decreased over getting score in R.I.R.S. score system (*P* < 0.001), group of this score (*P* < 0.001) and RUSS (*P* < 0.001, Table 2). The binary logistic regression analysis revealed that the R.I.R.S. scoring system and RIPA, lower pole stones, RIL, kidney stone density, and stone burden were considered as independent factors affecting the success of RIRS (*P*: 0.033, *P*: 0.001, *P*: < 0.001, *P*: 0.007, *P*: 0.001, *P*: 0.006). The binary logistic regression analysis results are shown in Table 3.

**Table 1:** The demographic and clinical data of the 147 patients and the stone characteristics used to calculate the R.I.R.S. score

	No. of the patient (%)	SF	Non-SF	*P* value
No. of the patient (%)	147	105 (71.43%)	42 (28.57%)	
Gender				0.949^a^
Male	116 (78.91%)	83 (71.55%)	33 (28.45%)	
Female	31 (21.09%)	22 (70.97%)	9 (29.03%)	
Age	52.00 (61.00–40.00; 21)	53.00 (59.50–38.50; 21.00)	50.00 (63.25–42.25; 21.00)	0.494^d^
Hypertension				0.544^a^
Yes	27 (18.37%)	18 (66.67%)	9 (33.33%)	
No	120 (81.63%)	87 (72.50%)	33 (27.50)	
Diabetes mellitus				0.120^a^
Yes	10 (6.80%)	5 (50.00%)	5 (50.00%)	
No	137 (93.20%)	100 (72.99%)	37 (27.01%)	
BMI (kg/m^2^)	24.39 (26.82–22.20; 4.62)	24.49 (27.12–22.67; 4.44)	23.66 (24.93–21.67; 3.26)	0.139^d^
History of surgery				0.088^a^
Yes	29 (19.73%)	17 (58.62%)	12 (41.38%)	
No	118 (80.27%)	88 (74.58%)	30 (25.42%)	
Laterality				0.085^a^
Left	78 (53.06%)	51 (65.38%)	27 (34.62%)	
Right	69 (46.94%)	54 (78.26%)	15 (21.74%)	
RIPA (°)	38.40 ± 23.03	43.70 ± 22.52	25.15 ± 18.75	< 0.001^c^*
Inferior pole stone				< 0.001^b^*
Yes	91 (61.90%)	53 (58.24%)	38 (41.76%)	
No	56 (38.10%)	52 (92.86%)	4 (7.14%)	
RIL (mm)	24.72 (27.31–20.95; 6.36)	24.00 (26.07–20.09; 5.98)	26.47 (28.05–23.39; 4.66)	0.005^d^*
RIW (mm)	8.15 (10.86–6.47; 4.39)	8.56 (10.70–6.52; 4.18)	7.48 (11.47–6.16; 5.31)	0.839^d^*
Stone burden (mm)	13.21(20.00–10.00;10.00)	12.21 (17.36–9.84; 7.52)	15.93 (25.00 –10.75;14.25)	0.003^d^*
Number of stone				0.352^a^
Single	106 (72.11%)	78 (73.58%)	28 (26.42%)	
Multiple	41 (27.89%)	27 (65.85%)	14 (34.15%)	
Renal stone density (Hu)	1403.19 ± 281.40	1330.59 ± 255.41	1584.69 ± 263.16	< 0.001^c^*
Urine culture				0.762^a^
Positive	36 (24.49%)	25 (69.44%)	11 (30.56%)	
Negative	111 (75.51%)	80 (72.07%)	31 (27.93%)	
R.I.R.S. score	7.39 ± 1.31	7.07 ± 1.18	8.19 ± 1.29	< 0.001^d^*

*BMI* Body mass index, *RIPA* renal infundibulopelvic angle, *RIL* renal infundibulopelvic length, *RIW* renal infundibular width

*Statistical significance was set at *P* < 0.05

^a^Chi-square test

^b^Fisher's exact test

^c^Student's t test

^d^Mann-Whitney U test

**Table 2:** The SFR after RIRS according to the R.I.R.S. scoring system and RUSS

R.I.R.S. score	SFR (%)	R.I.R.S. score group	SFR (%)	RUSS	SFR (%)
4	100% (1/1)	Mild (4–5)	90% (9/10)	0	85.00% (51/60)
5	88.89% (8/9)			1	70.91% (39/55)
6	86.67% (26/30)	Moderate (6–8)	80.19% (85/106)	2	72.22% (13/18)
7	81.08% (30/37)			3	14.29% (2/14)
8	74.36% (29/39)	Severe (9–10)	35.48% (11/31)		
9	41.67% (10/24)				
10	14.29% (1/7)				
Total	71.43% (105/147)				
Linear-by-linear association	*P* < 0.001		*P* < 0.001		*P* < 0.001

**Table 3:** Binary logistic regression analysis of potential independent predictors for postoperative stone-free outcomes

	*P* value	Exp (B)	95% CI
Inferior pole stone	< 0.001*	0.56	0.011–0.273
RIPA	0.001*	0.942	0.910–0.976
RIL	0.007*	1.165	1.042–1.303
Renal stone density	0.001*	1.004	1.002–1.006
Stone burden	0.006*	1.121	1.034–1.216
R.I.R.S. score	0.033*	0.438	0.205–0.935

*RIPA* renal infundibulopelvic angle, *RIL* renal infundibulopelvic length, *RIW* renal infundibular width, *CI* confidence interval

*Statistical significance was considered at *P* < 0.05

## Discussion

As we know, PCNL has always been the gold standard for the treatment of kidney stones larger than 20 mm [9], but in recent years, patients have rising need for non-invasive surgery. This is why more and more large medical centers are performing ultra-guided surgery.

RIRS is less invasive than PCNL and has a lower incidence of complications. RIRS has a higher stone removal rate and fewer additional operations than shockwave lithotripsy (SWL) [10]. The disadvantages of RIRS are its expense and the fragility of the ureteroscope, which adds to the cost of peripherals, including extraction baskets, laser fiber, and access sheaths. The best treatment should balance the benefits, potential complications, and total costs [11]. For this reason, accurate preoperative evaluation and preoperative planning of patient information are crucial.

The RUSS was summarized by Resorlu et al. in 2012 through a single-center retrospective analysis of 207 patients with kidney stones undergoing flexible surgery. Its parameters include stone size, number of stones, lower pole stones with RIPA < 45°, and abnormal renal anatomy (horseshoe kidney or pelvic kidney). Stone-free status is defined as an absence of stones or residual fragments ≤ 1 mm under computed tomography (CT) examination 1 month after surgery. The final total SFR = 86% [12]. This scoring system has been externally verified by many scholars, proving its effectiveness in predicting postoperative SFR [11]. This scoring system is the most widely used scoring system at present, but it was found that the stone composition is closely related to SFR during the research process. Because the stone’s composition cannot be known before the operation, this parameter is not included in the scoring system. However, some scholars discovered that the composition of stones is closely related to their density [13]. Therefore, to render this scoring system more accurate and reasonable, the density of stones should be used in scoring systems.

The R.I.R.S. score is a simple and comprehensive scoring system summarized by Xiao et al. in 2017 in a retrospective analysis of 382 kidney stone patients undergoing RIRS surgery. It contains four parameters, namely stone density, stone burden, RIL, and whether the stone is located in the lower calyx (if it is located in the lower calyx, RIPA should be considered). Stone-free status is here defined as no stones detectable under KUB, or only stones smaller than 2 mm 1 month after surgery. SFR = 73.6% [5]. The patients enrolled in this scoring system had undergone CTU examinations before surgery and performed three reconstructions, which can accurately measure all the stones’ data, including RIPA, RIW, RIL, and other renal anatomical structures. The total score can range from 4 to 10 points. The higher the score, the more complicated the stone and the lower the rate of stone clearance after surgery. It is a simple and comprehensive scoring system. However, many medical centers do not use CTU as a routine preoperative inspection. This may be one drawback of this scoring system; it cannot be widely promoted.

Establishing an accurate diagnosis and determining the best treatment and surgery plan, preoperative imaging is a crucial step [14]. In our center, patients undergo routine preoperative CTU examinations to better understand the patient’s urinary tract conditions and the characteristics of stones. NIKESH et al. suggested that CTU should be the standard imaging examination before PCNL because CTU can provide high-resolution spatial imaging and multiplanar reconstruction to accurately indicate the complexity, size, number and distribution of stones, renal pelvis structure and the anatomical relationship with other structures, and so reduce surgical complications and improve SFR and prevent unnecessary increases in the radiation burden [15]. Saskia et al. [16] suggest that CTU is vital before performing an endourologic procedure because CTU can show the anatomy of the renal pyelocaliceal system. Therefore, we recommend that the CTU examination is necessary before RIRS.

**Fig. 2:**
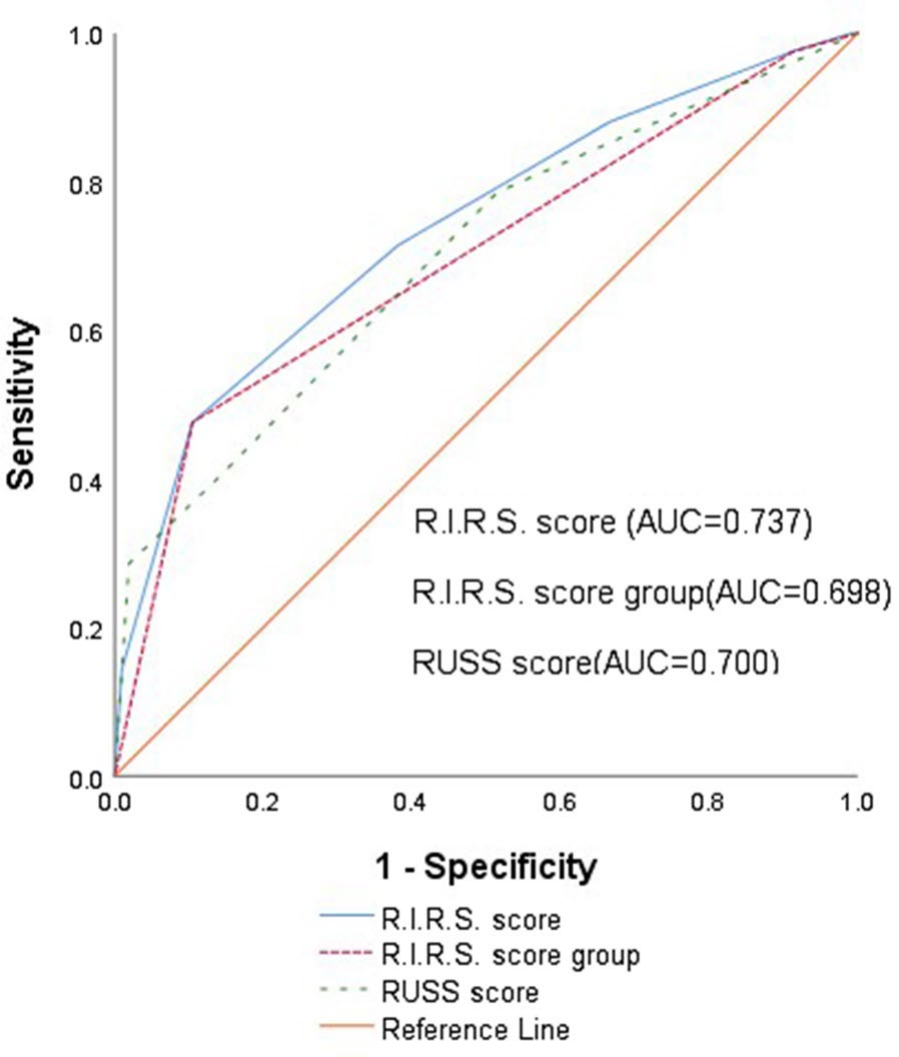
Receiver operating characteristic curves of the original and three-tiered R.I.R.S. score groups and RUSS

We found that the R.I.R.S. scoring system is easy to repeat because the CTU can evaluate all parameters. The RIPA is the most demanding parameter in the evaluation. In our experience, angles can be quickly and effectively assessed after a few learning curves. In this study, we propose evaluating the findings of Xiao et al. [5] and retrospectively validate the R.I.R.S. scoring system in 147 patients treated with RIRS at a single tertiary center by an experienced endourologist. We have found that the R.I.R.S. scoring system is an independent predictor of postoperative stone-free status. It provides high prediction accuracy (AUC = 0.737, Fig. 2).

R.I.R.S. scoring system seems to be a reliable preoperative tool for estimating the probability of stone-free status after RIRS. The practical value of this tool is mainly reflected in two points: patient consultation and research. By quantitatively estimating the complexity of stones, doctors can inform patients of the probability of successful treatment, and quantitative estimates can increase accurate data exchange between researchers.

To obtain a certain number of samples in a short time, this study chose to conduct a retrospective study, so it is inevitable that there are some limitations. First, the patient did not use a unified imaging method to detect the postoperative situation. To reduce the patient's radiation and economic burden, we generally use KUB to evaluate the patient's stone removal, and if there is suspected stone, further CT examinations are performed. This will lead to a certain error in the stone-free rate and lead to a certain degree of bias in the research results. Secondly, this study excluded patients who did not undergo CTU examination and patients who did not complete the entire operation, which may lead to selection bias. Finally, we are a single-center small sample size study. Our work's accuracy could not be compared with prospective, multicenter, and large-sample because of these limitations.

## Conclusion

This study retrospectively validates that R.I.R.S. scoring system is a simple system that may predict postoperative stone-free rate after RIRS, and can provide high prediction accuracy.

## Data Availability

The raw data used and analyzed in the study are available from the corresponding author on reasonable request. The data used in this study were anonymized before its use.

## Abbreviations

RIRS: Retrograde intrarenal surgery
SFR: Stone-free rate
RIPA: Renal infundibulopelvic angle
RIL: Renal infundibulopelvic length
CSD: Cumulative stone diameter
CTU: Computed tomography urography
NCCT: Non-contrast computed tomography
BMI: Body mass index
UAS: Ureteral access sheath
RIW: Renal infundibular width
CI: Confidence interval
KUB: Kidney ureter bladder
PCNL: Percutaneous nephrolithotomy
CT: Computed tomography
SWL: Shock wave lithotripsy
